# The effects of exercise on C-reactive protein, insulin, leptin and some cardiometabolic risk factors in Egyptian children with or without metabolic syndrome

**DOI:** 10.1186/1758-5996-4-27

**Published:** 2012-06-12

**Authors:** Nashwa Nabil Kamal, Merhan Mamdouh Ragy

**Affiliations:** 1 Lecturer of public health, Public health department, Faculty of Medicine, Minia University, Minia, Egypt; 2 Lecturer of physiology, Physiology department, Faculty of Medicine, Minia University, Minia, Egypt

**Keywords:** Metabolic syndrome, Exercise, Children, Cardiometabolic risk factor, C-reactive protein and insulin

## Abstract

**Background:**

The prevalence and magnitude of obesity in the children and the adolescents have increased dramatically in the developing countries over the last 20–30 years. The prevalence of metabolic syndrome (MS) in children is increasing. Aim: This study aimed to investigate the changes of C-reactive protein (CRP), leptin, insulin, and blood lipids before and after the exercise therapy in normal and obese children (with or without metabolic syndrome).

**Methods:**

The study covered 49 normal children (control), 32 obese children without metabolic syndrome and 12 obese children with metabolic syndrome. We examined the influence of exercise (3 times/week) for 12 weeks on the levels of serum CRP, leptin, insulin, homeostatic model assessment insulin resistance (HOMA-IR), triglycerides (TG), total cholesterol (TC), low-density lipoprotein cholesterol (LDL-C), and high-density lipoprotein cholesterol (HDL-C) in all groups.

**Results:**

There were significant correlations between HOMA-IR and the individual components of the metabolic syndrome. After 12 weeks of exercise, both of the obese children groups, with and without metabolic syndrome, showed reduced body weight, body mass index (BMI), and CRP level, and increased HDL-C level. The percentage of metabolic syndrome decreased from 12.9% before the exercise training to 7.5% after training. Also, there was a significant reduction in BMI (from 47.3 to 32.6%), in systolic blood pressure (from 18.3 to 15.1%) and in HDL-C level (from 18.3 to 9.7%).

**Conclusion:**

Overweight children have multiple risk factors associated with the metabolic syndrome. 12-week exercise may have a positive effect on reducing risk factors for the metabolic syndrome.

## Background

Metabolic syndrome is a cluster of cardiovascular disease risk factors that include glucose intolerance, hypertension, elevated TG, low HDL-C, and obesity [1]. This clustering has been shown to occur not only in adults but also in adolescents [2]. Although the metabolic syndrome is particularly important in adults, the pathological processes and risk factors have been shown to begin during childhood [3].

This syndrome continues to increase in both developed and developing countries, and has already become a major threat to the global public health [4].

The prevalence of the metabolic syndrome in the children and the adolescents is relatively low; about 4% [5]. When compared to the adult population (24%), except amongst the overweight and obese adolescents where the prevalence of the metabolic syndrome has been reported as high as 29% [6].

Obesity is strongly associated with elevated concentrations of circulating markers of inflammation, such as CRP [7]. Elevated plasma levels of CRP have been associated with an increased risk of coronary heart disease and type 2 diabetes [8]. Childhood CRP values predict adult CRP [9].

Leptin, a hormone secreted by adipose tissue, acts on the inhibiting food intake and stimulating energy expenditure [10]. Leptin increases in obesity, type 2 diabetes mellitus, hypertension and metabolic syndrome [11].

Exercise training programs can improve the insulin sensitivity, the vascular endothelium function, the glycemic control, and the blood pressure [12]. However, there have been few studies of changes of cardiovascular parameters with physical exercise programs in the obese children.

In Egypt, no study has examined the relationship between exercise and metabolic syndrome in the children. Therefore, the aims of this work were to:-

Evaluate the effect of physical training on BMI, lipid profile, CRP, leptin and insulin resistance among obese Egyptian children with and without metabolic syndrome.

Assess the relationship between HOMA-IR and the metabolic syndrome parameters.

## Methods

### Subject selection

Ninety three children of both sexes, aged from 8 to 12 years old, were included in this study. Three groups comprising:

1 Control normal weight group (n = 49) normal weight children with a BMI value between the 25^th^ and 74^th^ percentile.

2 Obese without metabolic syndrome group (n = 32).

3 Obese with metabolic syndrome group (n = 12).

### Obese children

are those with BMI greater than 95^th^ percentile for their age and gender. They were recruited in the nutritional clinic in the University Hospital by open invitation. Both the children and their parents were fully informed of the nature and purpose of the present study, and written informed consents were obtained. The experimental protocols were approved by the Medical Ethics Committee of Minia University and the investigations were conducted according to the principles expressed in the Declaration of Helsinki.

None of the subjects used drugs or therapies for obesity, and none had a past history of disease or injury that would prevent daily exercise. Children were currently participating in an organized physical activity training program over the previous 6 months, and were also excluded from the study.

### Diagnosis of metabolic syndrome

The criteria for the metabolic syndrome in adults developed by the National Cholesterol Education Program were modified and devised by many investigators [13-15]. as a child-specific definition which includes abnormalities in any three of the following factors: fasting glucose level (>110 mg/dL), TG level (>110 mg/dL), HDL-C level (<40 mg/dL), systolic or diastolic blood pressure (> 90^th^ percentile), and BMI (> 85^th^ percentile).

### Anthropometric measurement

Body weight and height were measured on the same day to the nearest 0.1 kg and the nearest 0.1 cm, respectively. The BMI was calculated as the weight in kilograms divided by the square of the height in meters. The waist circumference was measured to the nearest 0.1 cm, using a non-extendable flexible tape applied above the iliac crest and parallel to the ground; with the subject standing erect with abdomen relaxed, arms along the body, and feet together.

### Exercise training intervention

All children performed a set of exercises 3 days a week, for 12 weeks by a trained exercise physiologist. At each training session, they performed warm-up exercises for 5 – 10 min, followed by a 20 – 45 min walking–jogging exercise, with a target heart rate reserve of 60 – 65%, and relaxation exercises for 5 – 10 min at the end of the exercise period.

The heart rate reserve, used to calculate the intensity of exercise, was determined by counting heart beats at the carotid artery 10 s after the cessation of exercise and then using the Karvonen equation $\left[\left(heartratemaximum–heartraterest\right)\times \left(0.60–0.65\right)\right]+heartraterest$[16].

### Measurements and biochemistry

Blood samples were collected, via the cannulated antecubital vein, between 8:00–9:00 a.m. after an overnight fasting for all groups before and after the exercise program. Serum and platelet-poor plasma were obtained by centrifugation at 2000 g at a temperature of 4°C for 15 minutes. The samples were subsequently stored at - 80°C until assayed.

TC, TG and HDL-C were measured using an enzymatic colorimetric assay (Human Gesellschaft for Biochemica and Diagnostica). LDL was calculated from the TC, TG and HDL according to the method of Friedewald et al. [17].

(1) $$LDL-C\;\left(mg/dl\right)=TC-TG/5–HDL-C\text{.}$$

Plasma glucose was determined by a glucose oxidase method (Sclavo Diagnostics International SPI). CRP was measured by turbidimetry (biosystems). Plasma leptin was measured by the DSL (Webster, TX) 10 –23100 ACTIVE Human Leptin enzyme-linked immunosorbant ELISA, an enzymatically amplified “two step” immunoassay. Plasma insulin was measured by the DSL-10-1600– ACTIVE insulin enzyme - linked immunosorbant ELISA. Insulin resistance (IR) was calculated by the homoeostasis model assessment (HOMA-IR) index as: $\left[fasting\;insulin\left(\mu U/ml\right)\times fasting\;glucose\;\left(mmol/L\right)/22.5\right]$. To distinguish normal from impaired insulin sensitivity, HOMA-IR greater than 2.5 and 4.0 were the cut-off points used in children and adolescents, respectively [18].

#### Statistical analysis

The data were reported as mean ± SD, and analysed using the SPSS® statistical package, version 11.0 (SPSS Inc., Chicago, IL, USA) for Windows®.

We verified normal distribution of variables with a Kolmogorov–Smirnov test, and the parametric variables with skewed distribution were expressed as mean ± SD. For the nonparametric variables (leptin, CRP, and TG); the median, along with the minimum and maximum values, were expressed in the descriptive tables. The comparisons between the measurements of the parametric parameters were determined by paired and unpaired samples t test.

Statistically significant differences were tested with the nonparametric Kruskal-Wallis test for quantitative items, and with the nonparametric Wilcoxon test for paired observations.

Binary logistic regression analysis was performed to examine the relationship between obesity and metabolic syndrome components.

Pearson’s correlation coefficients were used to evaluate the correlations between HOMA-IR and the individual components of the metabolic syndrome. A *P*-value of < 0.05 was considered to be statistically significant.

## Results

Table 1 shows the clinical and biological characteristics of the study group. The mean age of boys and girls was 10.5 ±1.27 years and 10.05 ± 1.16 years respectively. The mean weight of boys and girls was 57.6 ± 8.4 and 55.4 ± 7.8 respectively.

**Table 1:** Clinical and biological characteristics of the studied population according to sex

**Girls (n = 40)**	**Boys (n = 53)**	**Characteristics**
10.05 ± 1.16	10.5 ± 1.27	Age (y)
55.4 ± 7.8	57.6 ± 8.4	Weight (kg)
146.3 ± 8.7	149.4 ± 8.8	Height (cm)
21.4 ± 6.1	22.7 ± 6.4	BMI (kg/m^2^)
70.2 ± 9.5	70.4 ± 10.6	Waist circumference
0.84 ± 0.05	0.83 ± 0.06	Waist-hip ratio
112.3 ± 7.7	114.8 ± 8.9	Systolic BP (mmHg)
74.6 ± 8.04	73.9 ± 6.9	diastolic BP (mmHg)
160 ± 24.9	161.5 ± 21.6	Total cholesterol (mg/dL))
98.4 ± 15.8	100.3 ± 13.9	LDL ± C (mg/dL)
45.8 ± 8.2	47.1 ± 8.6	HDL-C (mg/dL)
88 (75–135)	95 (72–150)	Triglyceride (mg/dL) †
85.9 ± 7.3	88.4 ± 10.9	Glucose (mg/dL)
15.3 ± 4.6	14.8 ± 4.9	Insulin ( IU/mL)
2.6 ± 0.8	2.7 ± 0.8	HOMA-IR
0.24 ( 0.01-2.5)	0.9 ( 0.01-2.6)	CRP (mg/L) †
5.7 (0.6-21.5)	6.5 (0.6-36.5)	Serum leptin (ng/mL) †

Data marked with † were presented as medians (interquartile ranges) and all others were presented as means ± standard deviations.

The distribution of five risk factors of the metabolic syndrome and the full syndrome with its odds ratio among the children are shown in Table 2. Low HDL-C was present in 34.1% of the obese vs. 4.1% of the normal-weight children (p < 0.001). Similarly for TG (25% vs. 8.2%) (p < 0.01) and high systolic blood pressure (27.3% vs. 10.2%) (p < 0.05). Metabolic syndrome was diagnosed in 27.3% of the obese children and none of the normal-weight children. Among the risk factors, low HDL-C gave the highest odds of 1.2 (95% CI 1.02, 1.3) of developing metabolic syndrome followed by high fasting blood glucose (OR = 0.9; 95% CI 0.86, 1.04), and high triglycerides (OR = 0.9; 95% CI 0.8, 0.9).

**Table 2:** Metabolic profile and metabolic syndrome components among the studied children according to sex

**Characteristic**	**Obese children**			**Control**			**OR (95%CI)**^**a**^
	**Females (n = 16) n (%)**	**Males (n = 28) n (%)**	**Total (n = 44) n (%)**	**Females (n = 24) n (%)**	**Males (n = 25) n (%)**	**Total (n = 49) n (%)**	
BMI > 85th percentile	16 (100)	28 (100)	44 (100)	0 (0)	0 (0)	0 (0)	-
Impairment of glucose tolerance	3 (18.7)	4 (14.3)	7 (15.9)	2 (8.3)	1 (4)	3 (6.1)	0.9 (0.86-1.04)
Systolic blood Pressure > 95th Percentile	5 (31.2)	7 (25)	12 (27.3)	2 (8.3)	3 (12)	5 (10.2)	0.8 (0.7-0.9) ^b^
HDL < 5th percentile	6 (37.5)	9 (32.1)	15 (34.1)	2 (8.2)	0 (0)	2 (4.1)	1.2 (1.02-1.3) ^d^
Triglycerides > 95th percentile	3 (18.7)	8 (28.6)	11 (25)	1 (4.2)	3 (12)	4 (8.2)	0.9 (0.8-0.9) ^c^
Metabolic syndrome	4 (25)	8 (28.6)	12 (27.3)	0 (0)	0 (0)	(0)	-

b significant at p < 0.05; c significant at p < 0.01; d significant at p < 0.001.

a OR calculated from logistic regression test by using enter method.

Children with metabolic syndrome had a significantly higher body weight, BMI and waist circumference compared to obese and control groups (Table 3). Serum insulin, glucose, diastolic blood pressure, TG, leptin and CRP were significantly higher in metabolic syndrome group compared to obese and control groups. TC, LDL-C and systolic blood pressure values were significantly higher in metabolic syndrome group, compared to control group. The body weight, BMI, waist circumference, systolic and diastolic blood pressure, as well as TC, LDL-C, TG, leptin, and CRP levels were significantly higher in the obese group without metabolic syndrome than in the control group (Table 3).

**Table 3:** Clinical and biological characteristics of the studied population with weight and metabolic syndrome status specification

	**Control children (n = 49) Mean ± SD**	**Obese children without metabolic syndrome (n = 32) Mean ± SD**	**Obese childrenwith metabolic syndrome (n = 12) Mean ± SD**
Age (y)	10.1 ± 1.21	10.2 ± 1.2	11.04 ± 1.15^a, b^
Weight (kg)	62.6 ± 4.1 ^C^	50.2 ± 3.7	67.1 ± 6.3 ^a, b^
Height (cm)	146.5 ± 10.3	147.6 ± 7.5	153.9 ± 8.5 ^a, b^
BMI (kg/m^2^)	27.01 ± 3.9 ^C^	17.2 ± 2.5	29.4 ± 4.8 ^a, b^
Waist circumference	77.3 ± 5.2 ^C^	62.7 ± 6.6	83.25 ± 2.8 ^a, b^
Waist-hip ratio	0.85 ± 0.06	0.83 ± 0.06	0.88 ± 0.05 ^b^
Systolic BP (mmHg)	117.5 ± 6.8 ^C^	109.4 ± 6.9	121.7 ± 7.8 ^b^
diastolic BP (mmHg)	75.5 ± 8.3 ^C^	71.8 ± 6.3	80.4 ± 4.5 ^a b^
Total cholesterol (mg/dL))	178.5 ± 16.8 ^C^	145.2 ± 13.6	177.7 ± 21.9 ^b^
LDL ± C (mg/dL)	107 ± 10.2 ^C^	92.6 ± 14.5	107.5 ± 12.1 ^b^
HDL-C (mg/dL)	45.8 ± 5.9 ^C^	50.5 ± 6.5	32 ±2.7 ^a, b^
Triglyceride (mg/dL)	95.5 (76–135) ^C^	85 (72–123)	118.5 (110–150) ^a, b^
Glucose (mg/dL)	84.9 ± 5.7	84.1 ± 5.2	105.5 ± 11.04 ^a, b^
Insulin ( IU/mL)	14.4 ± 3.9	14.4 ± 4.9	19.3 ± 4.4 ^a, b^
HOMA-IR	2.7 ± 0.9	2.4 ± 0.6	3.3 ± 0.88 ^a, b^
CRP (mg/L)	1.25 ( 0.01-2.6) ^C^	0.2 ( 0.01-1.5)	2.1 (1.2-3.1) ^a, b^
Serum leptin (ng/mL)	9.4 (0.6-36.5) ^C^	4.5 (0.6-12.4)	15.2 (8.3-21.8) ^a, b^

a = significant difference between obese children with and without metabolic syndrome.

b = significant difference between non obese children and obese children with metabolic syndrome.

c = significant difference between non obese children and obese children without metabolic syndrome.

There were significant correlations between HOMA-IR and the individual components of the metabolic syndrome (Table 4).

**Table 4:** Pearson Correlation Coefficients between HOMA-IR and the individual components of the metabolic syndrome

**Variables**	**r**	**p**
Triglycerides*	0.41	0.001
HDL-C	−0.20	0.05
Systolic blood pressure	0.28	0.005
BMI > 85^th^ percentile	0.22	0.03
Fasting glucose	0.29	0.004

HDL-C = high-density lipoprotein cholesterol; HOMA-IR = insulin resistance as assessed by homeostasis model. *Spearman rho correlation.

**Table 5:** Changes in clinical and biological characteristics among the studied groups undergoing 12 week exercise program

	**Normal weight (*n* = 49) Mean ± SD**		**Obese with out metabolic syndrome (*n* = 32) Mean ± SD**		**Obese with metabolic syndrome (*n* = 12) Mean ± SD**	
	**Baseline**	**Post-interventional**	**Baseline**	**Post-interventional**	**Baseline**	**Post-interventional**
Weight (kg)	50.2 ± 3.7	49.6 ± 3.7	62.6 ± 4.1	60.2 ± 4.3*	67.1 ± 6.3	60.9 ± 2.8*
BMI (kg/m^2^)	17.2 ± 2.5	16.8 ± 2.8	27.01 ± 3.9	24.8 ± 3.1*	29.4 ± 4.8	24.2 ± 2.2*
Waist circumference	62.7 ± 6.6	61.6 ± 5.9	77.3 ± 5.2	76.2 ± 5.6	83.25 ± 2.8	77.6 ± 5.2*
Waist-hip ratio	0.83 ± 0.06	0.80 ± 0.08	0.85 ± 0.06	0.83 ± 0.06	0.88 ± 0.05	0.85 ± 0.06
Systolic BP (mmHg)	109.4 ± 6.9	109.7 ± 7.1	117.5 ± 6.8	116.4 ± 7.2	121.7 ± 7.8	117.5 ± 6.9
diastolic BP (mmHg)	71.8 ± 6.3	71.6 ± 5.8	75.5 ± 8.3	72.2 ± 8.2	80.4 ± 4.5	77.1 ± 8.9
Total cholesterol (mg/dL))	145.2 ± 13.6	139.3 ± 15.1	178.5 ± 16.8	154.8 ± 20.7*	177.7 ± 21.9	166.3 ± 9.9
LDL ± C (mg/dL)	92.6 ± 14.5	91.7 ± 14.9	107 ± 10.2	99.4 ± 15.4*	107.5 ± 12.1	103.8 ± 15.7
HDL-C (mg/dL)	50.5 ± 6.5	51.9 ±5.4	45.8 ± 5.9	49.3 ±6.5*	32 ±2.7	43.4 ±6.2*
Triglyceride (mg/dL)	85 (72–123)	85 (72–112)	95.5 (76–135)	95.4 (76–124)	118.5 (110–150)	103.5 (89–135)*
Glucose (mg/dL)	84.1 ± 5.2	83.3 ± 5.6	84.9 ± 5.7	84.6 ± 5.5	105.5 ± 11.04	91.7 ± 10.8*
Insulin ( IU/mL)	14.4 ± 4.9	13.9 ± 4.9	14.4 ± 3.9	14.2 ± 4.6	19.3 ± 4.4	13.6 ± 4.01*
HOMA-IR	2.4 ± 0.6	2.4 ± 0.5	2.7 ± 0.9	2.5 ± 0.7	3.3 ± 0.88	2.7 ± 0.9
CRP (mg/L)	0.2( 0.01-1.5)	0.2 (0.01-1.5)	1.25( 0.01-2.6)	0.22(0.01-2.1)*	2.1 (1.2-3.1)	1.2 (0.04-2.25)*
Serum leptin (ng/mL)	4.5 (0.6-12.4)	3.8 (0.6-7.5)	9.4 (0.6-36.5)	5.7(0.6-16.9)*	15.2 (8.3-21.8)	6.1 (0.6-21.5)

*P < 0.05 for baseline vs after 12 weeks of physical exercise program within each group.

Table 5 shows that obese participants with metabolic syndrome showed significant reductions in weight, BMI, waist circumference, TG, glucose, insulin and CRP, and an increase in HDL-C after 12 weeks of training. The obese participants without metabolic syndrome presented significant reductions in weight, BMI, TC, LDL-C, CRP and leptin, and an increase in HDL-C in response to training.

**Figure 1:**
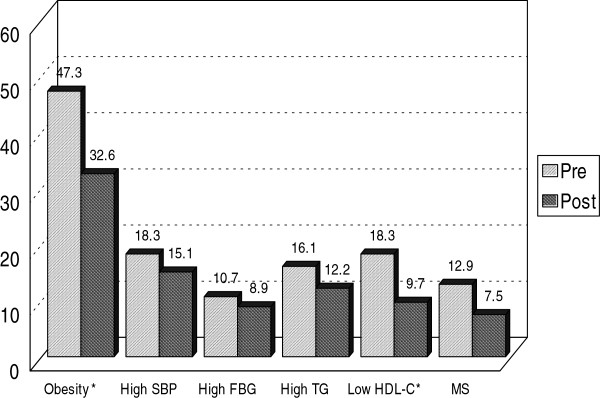
Variables in metabolic syndrome components before and after exercise intervention in all exercisers (n = 93).

The analysis of changes in parameters of metabolic syndrome is summarized in Figure 1. The percentage of metabolic syndrome decreased from 12.9% before the exercise training to 7.5% after training. Also, there was a significant reduction in BMI (from 47.3 to 32.6%), systolic blood pressure (from 18.3 to 15.1%), and in HDL-C (from 18.3 to 9.7%).

## Discussion

Metabolic syndrome was diagnosed in 12 (27.3%) of the total number of obese children, most of them correspond to the male group. It is slightly lower than a previous study [19]. which reported that metabolic syndrome with obesity occurs in 38.7% of moderately obese and 49.7% of severely obese children and adolescents.

The relationship between obesity and CRP levels in the children is limited, especially among the children younger than 12 years of age [20]. In the present study, CRP concentrations were elevated predominantly in obese individuals who were also insulin resistant and fall in parallel with weight loss–associated improvements in IR. CRP levels increased significantly in the group of obese children with or without metabolic syndrome compared to control group. Obese with metabolic syndrome group had higher CRP and insulin level in comparison with obese without metabolic syndrome group. This finding was in agreement with a previous study [21]. which found positive significant correlation between CRP, BMI, insulin level and IR. The mechanisms responsible for the association between CRP, BMI and IR are not yet clearly understood. It is possible that with increasing BMI, adipose tissue is a direct source of pro-inflammatory cytokines such as tumour necrosis factor-alpha (TNF-α) which, in turn, act as stimuli for CRP synthesis in the liver [22]. This leads to a chronic inflammatory state and contributes to IR [23].

In the present study the metabolic syndrome group had a greater TG and a lower HDL-C concentration similar to previous reports [24,25]. There were significant correlations between HOMA-IR and the individual components of the metabolic syndrome, including TG. IR is associated with decreased lipoprotein lipase activity, resulting in decreased clearance of TG, as well as increased lipolysis in adipose tissue and increased synthesis of very-low-density lipoprotein particles in the liver [26]. We found that exercise decreased TG and increased HDL-C by reversal of this effect.

Several physiological mechanisms exist to explain the changes in CRP and IR with exercise. Improvements in IR have been observed in adults upon participation in exercise programs; however, similar studies in overweight and obese children are limited [27,28]. Exercise is known to increase insulin-receptor autophosphorylation, glucose transporter 4 expressions and glucose transport [29]. Exercise increases the uptake of glucose by muscle cells which, consequently, increases the oxidation of glucose which leads to improvement in IR [30]. Exercise decreased CRP as reported in studies in adults reported by [31]. Exercise training reduces CRP both directly by reducing cytokine production in fat, muscle, and mononuclear cells, and indirectly by increasing insulin sensitivity, improving endothelial function, and reducing body weight [32].

The most significant effects of exercise on metabolic syndrome parameters are observed in HDL-C. This is in agreement with a previous study [33] which reported that the effects of exercise on HDL-C levels are most clearly seen in overweight persons with high TG levels and low HDL-C levels at baseline. In contrast, lean subjects with isolated low HDL-C levels had no significant increase in HDL-C with exercise, even though their lipoprotein lipase activity was increased. This suggests that the changes in HDL-C and LDL-C may be secondary to improvements in hypertriglyceridemia, IR, and adiposity. Other mechanisms for changes in HDL-C include increases in lipoprotein lipase activity and decreases in hepatic lipase and cholesteryl ester transfer protein [34,35].

Previous studies confirmed a positive relation between increased BMI and leptin levels in children and adolescents [36-38]. The results obtained in the present study demonstrated that leptin decreased significantly with exercise in obese without metabolic syndrome group, and this is in agreement with another study [39].

Obese children are associated with arterial endothelial dysfunction, which elevated blood pressure [40]. Exercise decreased both systolic and diastolic blood pressures. Potential mechanisms responsible for this change may include increased endothelial nitric oxide synthase activity and increased insulin sensitivity [41].

The present study had limitations, including that this project focused on increasing physical activity and no dietary intervention or outcomes were measured. Another limitation of the current study was the measurements of others inflammatory markers, such as TNF-alpha and IL-6, which would further increase the validity of the results, were not assessed due to high cost.

## Conclusions

Obese children exhibit several risk factors for the development of cardiovascular diseases and that metabolic syndrome is already a reality for many children, being present in 27.3% of the obese children investigated here. Exercise intervention reduces CRP, may be effective in preventing the occurrence of cardiovascular events. Regular physical activity is one of the most important non pharmacologic tools in reducing the overall cardiometabolic risk via regulating the body weight, BP and the lipid profile. Further studies will be required to assess the responses related to the degree of obesity, as well as the intensity and the duration of intervention.

## Abbbreviations

MS: Metabolic syndrome; CRP: C-reactive protein; TG: Triglycerides; HDL-C: High-density lipoprotein cholesterol; TC: Total cholesterol; LDL-C: Low-density lipoprotein cholesterol; HOMA-IR: Homeostatic model assessment insulin resistance.; TNF-alpha: Tumor necrosis factor alpha; BMI: Body mass index.

## Competing interests

No conflict of interest has been declared.

## Authors’ contributions

Author’s contribution to the paper is about equal. NNK collected data from the children, participated in the concept and design of the study, made the statistical analysis and wrote the manuscript. MMR designed and performed some biochemical kits measurements, contributed to the discussion and wrote the manuscript. All authors read and approved the final manuscript.
